# Transcriptomic analysis of the response of *Pseudomonas fluorescens* to epigallocatechin gallate by RNA-seq

**DOI:** 10.1371/journal.pone.0177938

**Published:** 2017-05-17

**Authors:** Xiaoxiang Liu, Bimiao Shen, Peng Du, Nan Wang, Jiaxue Wang, Jianrong Li, Aihua Sun

**Affiliations:** 1 Faculty of Basic Medicine, Hangzhou Medical College, Hangzhou, Zhejiang, P.R. China; 2 Department of Laboratory Medicine, Hangzhou Medical College, Hangzhou, Zhejiang, P.R. China; 3 College of Biology and Environmental Engineering, Zhejiang Shuren University, Hangzhou, P.R. China; 4 Food Safety Key Lab of Liaoning Province, Bohai University, Jinzhou, Liaoning, China; Rutgers, the State Univesity of New Jersey, UNITED STATES

## Abstract

Epigallocatechin gallate (EGCG) is a main constituent of green tea polyphenols that are widely used as food preservatives and are considered to be safe for consumption. However, the underlying antimicrobial mechanism of EGCG and the bacterial response to EGCG are not clearly understood. In the present study, a genome-wide transcriptional analysis of a typical spoilage bacterium, *Pseudomonas fluorescens* that responded to EGCG was performed using RNA-seq technology. A total of 26,365,414 and 23,287,092 clean reads were generated from *P*. *fluorescens* treated with or without 1 mM EGCG and the clean reads were aligned to the reference genome. Differential expression analysis revealed 291 upregulated genes and 134 downregulated genes and the differentially expressed genes (DEGs) were verified using RT-qPCR. Most of the DGEs involved in iron uptake, antioxidation, DNA repair, efflux system, cell envelope and cell-surface component synthesis were significantly upregulated by EGCG treatment, while most genes associated with energy production were downregulated. These transcriptomic changes are likely to be adaptive responses of *P*. *fluorescens* to iron limitation and oxidative stress, as well as DNA and envelope damage caused by EGCG. The expression of specific genes encoding the extra-cytoplasmic function sigma factor (PvdS, RpoE and AlgU) and the two-component sensor histidine kinase (BaeS and RpfG) were markedly changed by EGCG treatment, which may play important roles in regulating the stress responses of *P*. *fluorescens* to EGCG. The present data provides important insights into the molecular action of EGCG and the possible cross-resistance mediated by EGCG on *P*. *fluorescens*, which may ultimately contribute to the optimal application of green tea polyphenols in food preservation.

## Introduction

Contamination by pathogenic and spoilage bacteria is a major issue during food processing. To date, many synthetic and natural food additives have been used to control bacterial contamination and to extend the shelf-life of foods. With the increasing demands for safe and high-quality food, the development and utilization of effective natural preservatives are desirable. Green tea polyphenols (GTPs), key ingredients of green tea, have shown effective antimicrobial and antioxidant activities, and non-toxicity in the food industry [1]. In addition, GTPs are beneficial to health and are proposed to prevent cancer and several other chronic diseases [1–2]. Moreover, GTPs are ubiquity, abundance and low cost. So the application of GTPs in the food industry has attracted more interest, especially in fish or meat products. For examples, dip treatment with 0.2% GTP was effective in inhibiting spoilage bacteria growth and extending the ice storage life of fish samples to 35 days compared to 28 days for the control group [3]. The total viable counts reduced two orders of magnitude in *Collichthys* fish balls supplemented with 0.25 g/kg GTPs compared with the controls [4]. GTPs were also used combined with other natural preservatives. Application of GTPs (0.3% or 0.15%) in combination with 6-gingerol (0.3% or 0.15%) inhibited oxidation of protein and lipids, and reduced microorganism counts compared to control treatments during storage of shrimp paste [5]. The combination of nisin (0.625 g/L), GTPs (0.313 g/L) and chitosan (3.752 g/L) could be used as preservatives to efficiently inhibit the growth of spoilage microorganisms and pathogens in chilled mutton [6]. Although GTPs were considered to be a good choice of natural food preservatives, there were some challenges of the utilisation of GTPs in foods, such as the astringent and bitter taste [7], unstability during thermal processing and alkaline solutions [8–9], which may limit the application of GTPs in foods.

The most abundant components of GTPs are catechins, of which epigallocatechin gallate (EGCG) is the major one (50–80% of the total catechin content) [10]. GTPs and EGCG have a broad antimicrobial spectrum and inhibit the growth of many foodborne pathogenic and spoilage bacteria [4, 11]. Several studies have indicated that the antibacterial activity of EGCG is due to damage to the bacterial cell membrane, such as peptidoglycan [12], outer membrane proteins [13] and lipid bilayers [14]. Moreover, numerous key enzymes in bacterial cells have been suggested as the targets of EGCG, including FabG and FabI reductases [15], the DNA gyrase B subunit [16], thioredoxin, and thioredoxin reductase [17]. Instead of directly binding to protein targets, EGCG was reported to inhibit bacterial growth by producing H_2_O_2_ [18–19]. Most polyphenol compounds are effective metal chelators [20], and metal chelation may contribute to antibacterial activities [21]. In addition, green tea polyphenols and EGCG are able to disrupt biofilm formation and quorum sensing of bacteria [22–23]. Overall, the antibacterial action of EGCG may act in multiple ways and the complete mechanism has yet to be fully elucidated.

Modern food safety measures are designed only once there is a complete understanding of how foodborne microorganisms cope with stress conditions that are encountered in a variety of food products. Microorganisms can become resistant to otherwise lethal stresses due to exposure to sub-lethal stresses, especially when minimal food processing is involved, which creates challenges in developing new food processing techniques [24]. Although green tea polyphenols are widely used as food preservatives and are generally considered to be safe, cross-resistance can be induced by using green tea polyphenols and EGCG. Previously, EGCG-adapted strains of *Staphylococcal* have adapted increased resistance to antibiotics and heat stress [25]. Recently, short exposure of *Pseudomonas aeruginosa* to sub-lethal doses of green tea polyphenols or EGCG led to cross-resistance with some environmental stresses, including oxidants, various organic acids, salt, heat, ethanol and crystal violet [26]. Microorganisms often respond to environmental stress conditions by producing specific enzymes or proteins that aid in coping with various stresses [24]. Interestingly, expression of antioxidative genes is likely to be involved in the cross-resistance induced by green tea polyphenols in *P*. *aeruginosa* [19]. Proteomic analysis of *Escherichia coli* that has been exposed to green tea polyphenols indicates that several stress-related proteins were upregulated, such as the chaperone protein HSP60, RNA polymerase sigma factor RpoS, superoxide dismutase SodC and multidrug resistance efflux pump protein EmrK [27]. Additionally, short exposure to sublethal EGCG concentrations results in increased cell wall thickness, and the two-component VraSR system is suggested to be involved in modulating *Staphylococcus aureus* response to EGCG [25, 28]. Nevertheless, knowledge regarding how microorganisms respond to EGCG is still very limited.

*Pseudomonas fluorescens* is a common gram-negative spoilage bacterium that is widely found in food matrices, such as ready-prepared fresh vegetables [29], raw fish (especially sushi or sashimi) [30], meat and dairy products [31]. Due to its extreme adaptability, versatility and ability to replicate at refrigeration temperatures, long periods of shelf life can easily lead to increased *P*. *fluorescens* concentration in food products [4, 32]. Genome-wide transcriptomic analysis is an effective method in studying adaptive responses to the environment by identifying and linking associated transcriptional perturbations. However, there are currently few transcriptome analysis reports that describe the impact of polyphenols on bacteria [33]. In the present study, we exploited the global transcriptomic analysis of adaptive responses of *P*. *fluorescens* to EGCG by RNA-seq in order to further understand the antibacterial mechanisms of EGCG and the EGCG mediated cross-resistance in bacteria, which may ultimately contribute to the optimal application of green tea polyphenols in food preservation.

## Materials and methods

### Bacterial strains and media

The *P*. *fluorescens* strain ZJL511 was isolated previously from spoiled refrigerated turbot (*Scophthalmus maximus*). The bacterium was cultured in nutrient broth (NB; 1% peptone, 0.3% beef extract, 0.5% NaCl, pH 7.5) or plate count agar (PCA; 0.5% peptone, 0.25% yeast extract, 0.1% glucose, 1.55% agar, pH 7.0) at 30°C.

### Antibacterial activity of EGCG to *P*. *fluorescens*

*P*. *fluorescens* was cultured in NB medium at 30°C for 16 h and the cell culture then was diluted 1000-fold with NB medium (approximately 10^6^ CFU (colony forming units) /mL). The diluted *P*. *fluorescens* cell suspensions were cultivated in the presence (0.25, 0.5, 1, 2, and 4 mM) and absence of EGCG (Sigma, USA) at 30°C while shaking at 220 rpm. After 4 h of incubation, cells were diluted appropriately and poured in plate count agar. The cultures without EGCG treatment were used as controls. Viable cell numbers were determined by counting the colonies after 24–48 h of incubation at 30°C.

### EGCG exposure and RNA extraction

One millilitre of an overnight *P*. *fluorescens* culture was inoculated into 100 mL fresh NB medium (pH 7.5) and was incubated at 30°C with shaking at 220 rpm. At an OD_600_ of 0.4 (logarithmic phase), the culture was immediately treated with 1 mM EGCG. Culture untreated with EGCG was used as the control. After 1 h of exposure, the cells were harvested by centrifugation at 7,000 *g* for 10 min and were frozen in liquid nitrogen prior to RNA isolation. Total RNA was isolated from the frozen cells using TRIzol^®^ Plus RNA Purification Kit (Invitrogen, USA) according to the manufacturer’s instructions. Three independent biological replicates were performed for each treatment and the extracted RNA samples were mixed equally for each treatment. Total RNA was treated with RNase-Free DNase Set (Qiagen, Germany) to remove contaminating DNA. The quantity and quality of total RNA were assessed using a NanoDrop ND-2000 Spectrophotometer (Thermo, USA) and Agilent 2100 Bioanalyzer (Agilent, USA).

### Library preparation and sequencing

Strand-specific RNA sequencing libraries were prepared by following a modified deoxy-UTP (dUTP) strand-marking protocol described previously [34]. Briefly, total RNA from each sample was depleted of rRNA using a Ribo-Zero rRNA removal kit for Gram-negative bacteria (Epicentre, USA) per the manufacturer’s protocol, followed by RNA fragmentation. First-strand cDNA was synthesized using a hexamer primer (Takara, Japan) and Superscript II reverse transcriptase (Invitrogen, USA). Double-stranded cDNA was synthesized with dUTP incorporation into the second strand. The double-stranded cDNA fragments were further processed to construct a sequencing library. Prior to final amplification, the dUTP-marked strand was selectively degraded with USER enzyme (NEB, USA) and the remaining strand was amplified to generate a cDNA library suitable for sequencing. Following validation with an ABI StepOne Plus Real-Time PCR System (ABI, USA), the cDNA library was sequenced on a flow cell using high-throughput 125-bp pair-end mode on an Illumina HiSeq 2500V4 platform (Illumina, USA).

### Quality control and alignment

Clean data (clean reads) were obtained by removing adapter-containing, poly-N, and low-quality reads from the raw data (raw reads) using in-house Perl scripts. Meanwhile, the Q20, Q30 and GC content were calculated. All downstream analyses were based on clean data with high-quality. The genome sequence of *P*. *fluorescens* UK4 was used as the reference genome [35]. For each sample, sequence alignment with the reference genome sequences was performed using Tophat 1.3.1 [36]. The unique mapped reads were used in subsequent analyses.

### Analysis of differentially expressed genes (DEGs)

For gene expression analysis, the python script rpkmforgenes.py (last modified 13 November, 2014) was used to estimate the expression level (relative abundance) of specific transcripts expressed using the RPKM (Reads Per Kilobase per Million reads mapped) method [37]. The RPKM value was directly used for comparing differences in gene expression among samples, as this method eliminates influences due to different gene lengths and sequencing discrepancies in calculating gene expression. The software package edgeR 3.12.0 was used to calculate the fold-change of transcripts and to screen all differentially expressed genes (DEGs). The criteria of significant difference expression were |log_2_ fold change| ≥ 1 and False Discovery Rate (FDR) ≤ 0.05 [38–39].

### Cluster of orthologous group (COG) analysis for DEGs

DEGs functional description was performed using a *P*. *fluorescens* UK4 genome annotation and GO database. COG functional categories for the DEGs were obtained from the NCBI COG database (http://www.ncbi.nlm.nih.gov/COG/).

### DEG verification using RT-qPCR

In order to confirm the RNA-seq results, 17 upregulated and 5 downregulated genes from the RNA-seq analysis were selected and RT-qPCR was performed to confirm the gene expression changes of these 22 genes based on functional categories. *P*. *fluorescens* was treated with 1 mM EGCG and three biological replicates were collected. In addition, the expression levels of 4 representative upregulated genes were detected by RT-qPCR after *P*. *fluorescens* was treated with 0.5, 1 and 2 mM EGCG. Total RNA was isolated from each sample and reversely transcribed with a hexamer primer and SuperScript^™^ III First-Strand Synthesis SuperMix for RT-qPCR (Invitrogen, USA) according to the manufacturer’s instructions. The resulting cDNA was then used as the template for qPCR. Gene-specific primers were designed using the Primer Premier 6.0 and Beacon designer 7.8 software, while the 16S *rRNA* gene was used as the internal control (S3 Table). The reactions were performed using a CFX384 Touch^™^ Real-Time PCR Detection System (Bio-Rad, USA) with Power SYBR^®^ Green PCR Master Mix (Applied Biosystems, USA). The two-step qPCR program began at 95°C for 1 min, followed by 40 cycles of 95°C for 15 s and 63°C for 25 s. Fluorescent signals were collected at each polymerization step. Melting curve analysis of amplification products was performed at the end of each PCR program to confirm that a single PCR product was detected. Samples were assayed in triplicate and PCR reactions without the template were performed as negative controls. The comparative Ct method for relative quantification (ΔΔCt method) was used to analyze the data [40].

## Results and discussion

### Global overview of the RNA-seq data

**Fig 1:**
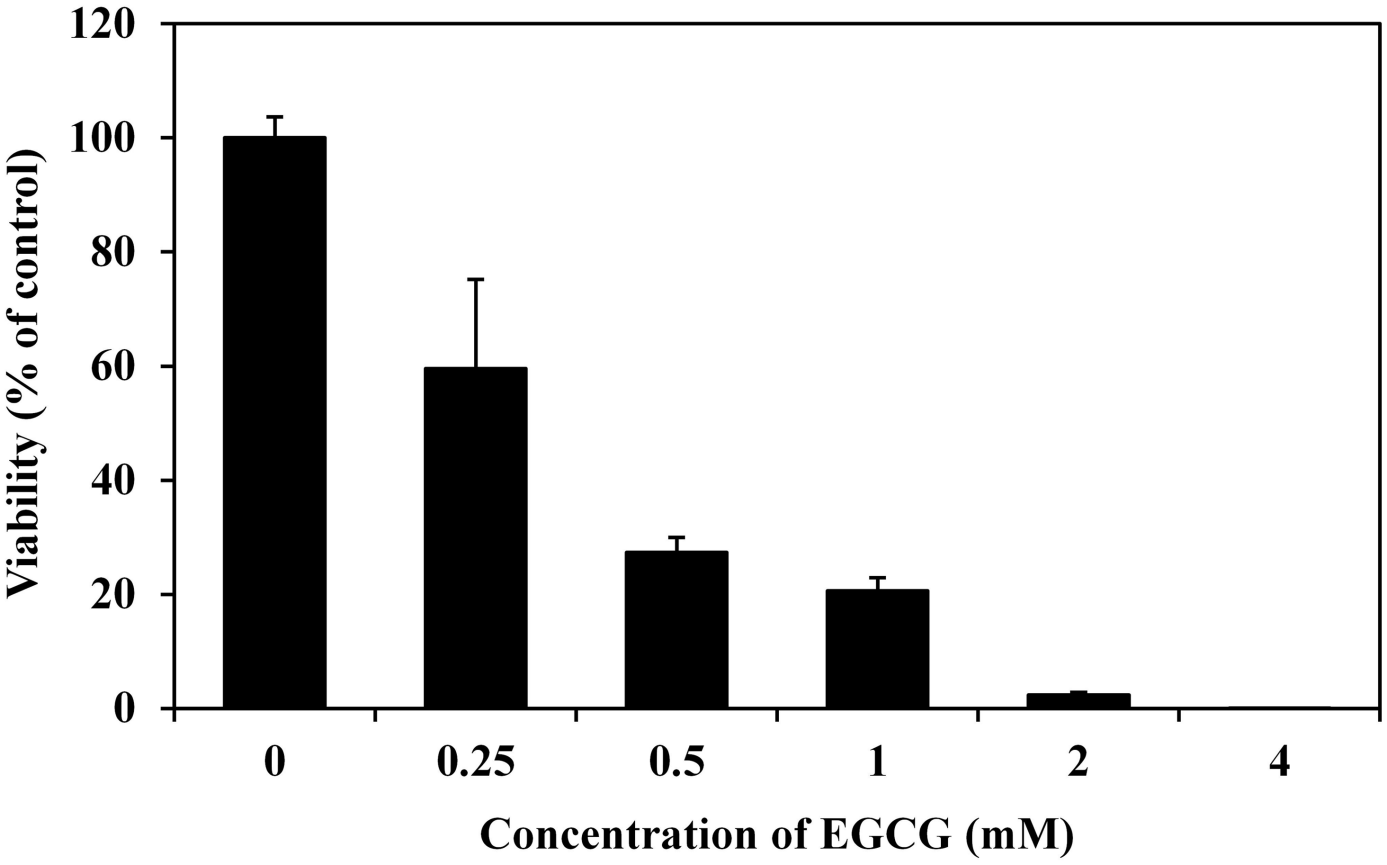
The antimicrobial action of EGCG against *P*. *fluorescens*. Different EGCG concentrations were supplemented to *P*. *fluorescens* cell suspensions (~10^6^ CFU/mL in NB medium, pH 7.5). After incubation at 30°C for 4 h with shaking at 220 rpm, cell viability was determined. Cell viability is expressed as a percentage of the control. Data are mean ± standard deviation from three independent experiments.

Prior to RNA-seq, the antibacterial action of EGCG against *P*. *fluorescens* was tested and EGCG had significant antibacterial activity that was dose dependent (Fig 1). Treatments of *P*. *fluorescens* with 0.25, 0.5, 1, 2 and 4 mM EGCG for 4 h significantly decreased survival rates (59.60%, 27.34%, 20.65%, 2.35% and 0.01% vs. control, respectively), demonstrating comparable antibacterial activity against *P*. *aeruginosa* as previously reported [19]. In order to avoid complete suppression of cellular metabolism, a moderate inhibiting concentration (1 mM) was selected for treatment in investigating the transcriptomic response of *P*. *fluorescens* to EGCG.

RNA-seq is an attractive method for monitoring global transcriptomic changes and overcomes many of the defects related to traditional DNA microarrays [41]. RNA-seq using the Illumina paired-end sequencing technology was used to examine the effect of EGCG on the *P*. *fluorescens* transcriptome. After filtering through the raw reads, there were 26,365,414 clean reads for the control sample and 23,287,092 for the EGCG-treated sample, giving rise to total clean bases of 3.30 G and 2.92 G, respectively (S1 Table). The clean sequencing data were deposited to the Short Reads Archive of NCBI with accession numbers SAMN05559903, SAMN05559904. Of the clean reads, the Q20 and Q30 values were > 92.68% and 85.68%, respectively, indicating that the data were of high quality. Among the *P*. *fluorescens* strains with complete genome sequence in GenBank, UK4 strain had the highest 16S gene homology with the test strain ZJL511 (data not shown), and thus the *P*. *fluorescens* UK4 strain sequence was used as the reference genome [35]. The clean reads of *P*. *fluorescens* transcriptome were compared to the reference genome sequence and the total mapped rates of the reads to the reference genome were 57.00% in the control sample and 58.00% in the EGCG group. There were 61.41% of the total genes in the reference genome detected (read number ≥ 1) by RNA-seq in the control, while there were 62.57% detected in the treated sample (S2 Table). The mapping rates were not high, which was likely due to high variability among the *P*. *fluorescens* genomes isolated [42]. For example, comparisons of three *P*. *fluorescens* genomes (SBW25, Pf0-1, Pf-5) revealed considerable divergence, showing that only 61% of genes were shared [43].

### Analyses of DEGs

A total of 425 DEGs were identified using the gene expression levels calculated by RPKM and the statistical criteria (|log_2_ fold change| ≥ 1, FDR ≤ 0.05). Among these genes, 291 genes were markedly upregulated and 134 genes were markedly downregulated in *P*. *fluorescens* following EGCG treatment (S1 Fig). The gene locus tags, log_2_ FC (fold change), FDR, RPKM, COG categories, gene product description and other data of the DGEs are provided in S4 and S5 Tables.

**Fig 2:**
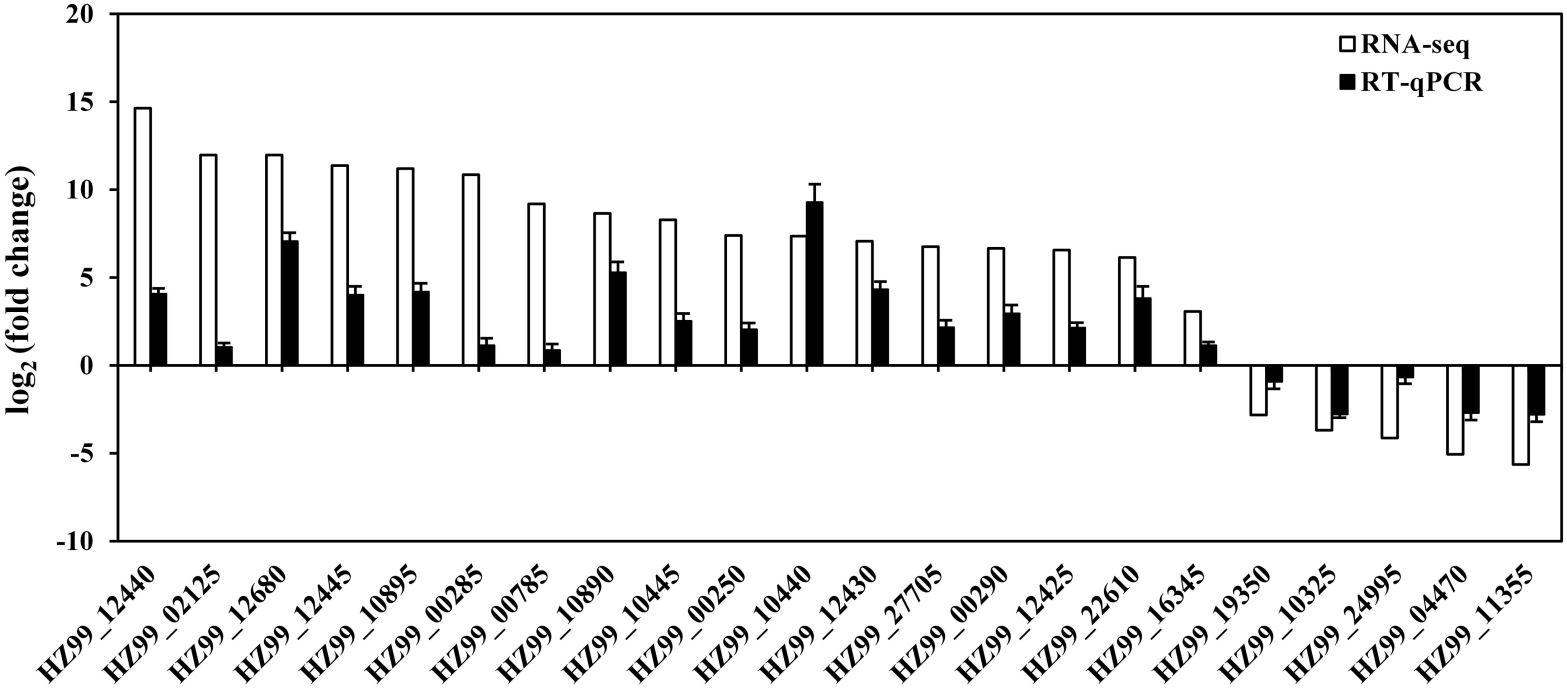
Validation of RNA-seq data using RT-qPCR. White bars represent RNA-seq data, while the black bars represent mean values of log_2_ (fold change) observed for EGCG-treated samples vs controls. Three biological replicates were performed for qRT-PCR. Error bars represent standard deviation.

**Fig 3:**
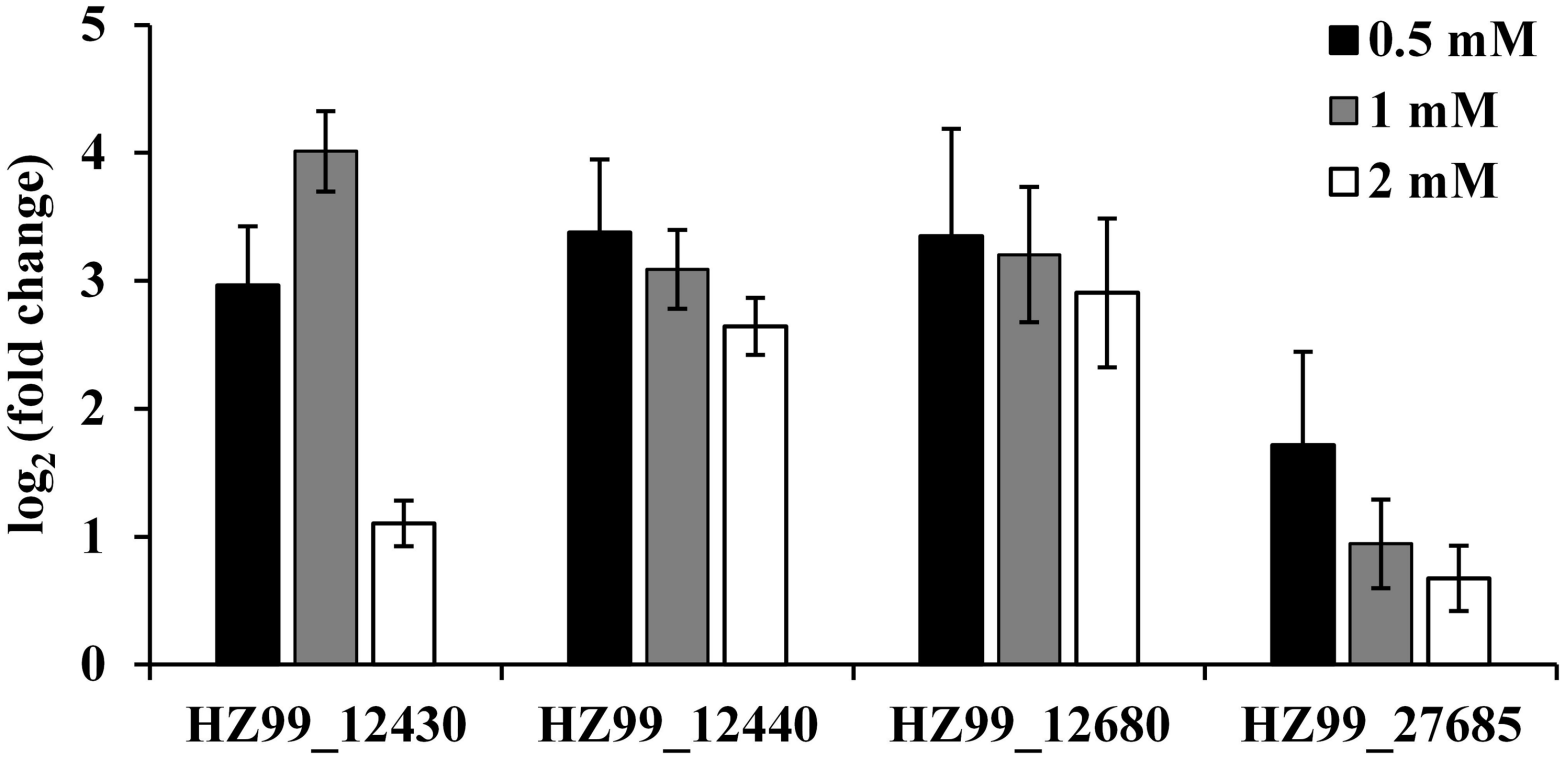
Expression analysis of DGEs after treatments with different concentrations of EGCG in *P*. *fluorescens*. White, gray and white bars represent 0.5, 1 and 2 mM EGCG treatment, respectively. The Y-axis shows values of log_2_ (fold change) observed for EGCG-treated samples vs controls. Three biological replicates were performed for qRT-PCR. Error bars represent standard deviation.

In order to validate the data generated from the RNA-seq experiment, 17 upregulated and 5 downregulated genes were selected from DGEs based on functional categories to verify their expression by RT-qPCR. All of the genes examined were consistent with the RNA-seq results (Fig 2), although the observed fold changes differed between qRT-PCR and RNA-seq data, which may reflect differences in the sensitivity and specificity between RT-qPCR and high-throughput sequencing technology. In addition, the expression levels of 4 representative upregulated genes were detected after 0.5, 1 and 2 mM EGCG treatment by RT-qPCR (Fig 3). The expressions of the 4 genes could be upregulated by each of the EGCG concentrations. These results suggested that the RNA-seq results were generally reliable.

**Fig 4:**
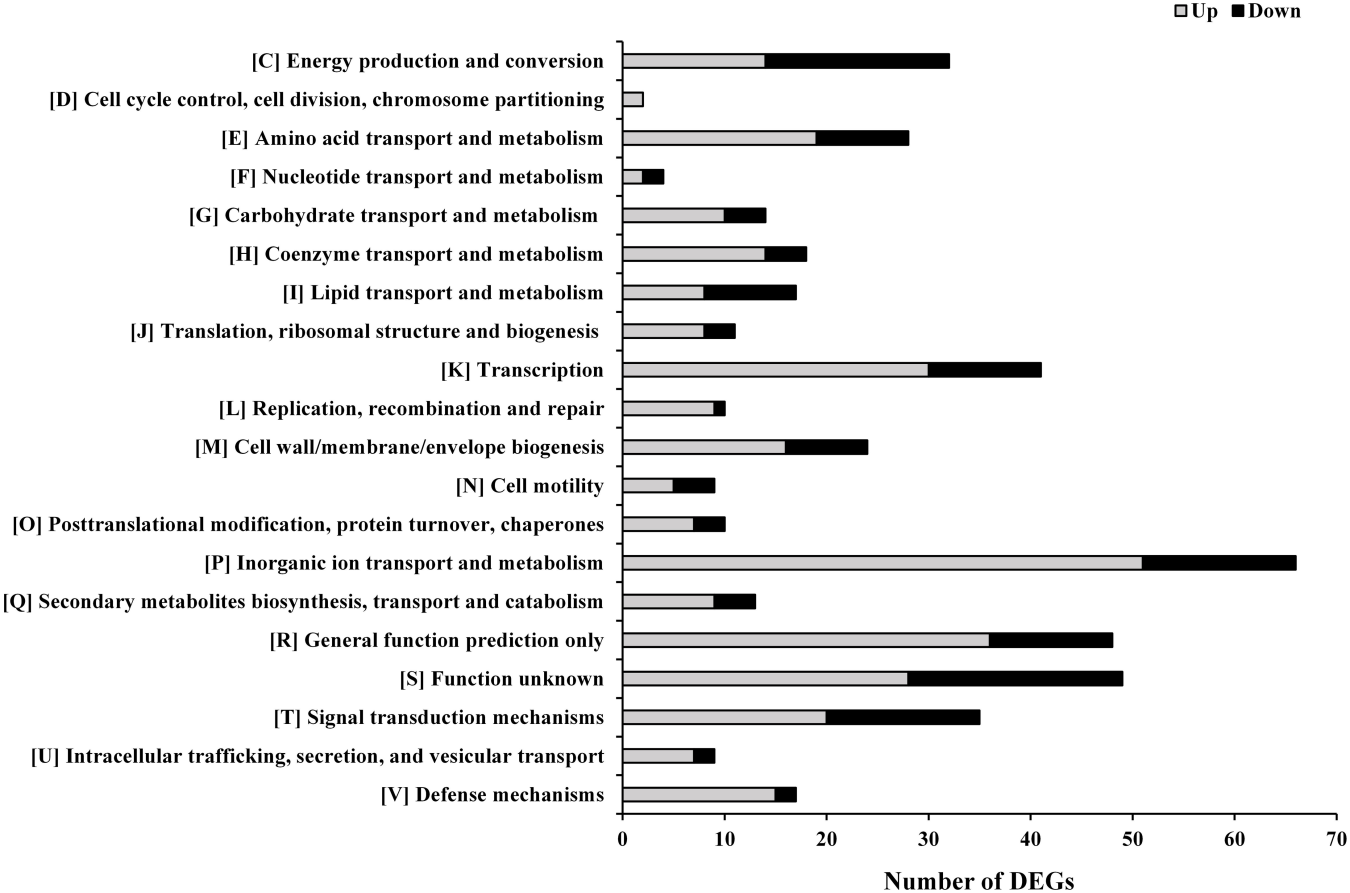
Classification of the upregulated and downregulated genes according to the COGs.

The 425 DGEs were classified using the known function of the gene products or through homology with protein functions determined from the COG database (Fig 4). COG functional analysis assigned DEGs to 20 predicted pathways, most of which belonged to the following groups: inorganic ion transport and metabolism (COG P; 15.53%), function unknown (COG S; 11.53%), general function prediction only (COG R; 11.29%), transcription (COG K; 9.65%), signal transduction mechanisms (COG T; 8.24%), energy production and conversion (COG C; 7.53%), amino acid transport and metabolism (COG E; 6.59%), cell wall/membrane/envelope biogenesis (COG M; 5.65%), coenzyme transport and metabolism (COG H; 4.24%), lipid transport and metabolism (COG I; 4.00%), and defense mechanisms (COG V; 4.00%). Moreover, in most of the COGs, a significantly larger number of the genes were upregulated rather than downegulated in the EGCG-treated sample versus the control (Fig 4). Only in COG C and COG I were more genes downregulated, and this was especially notable in COG I.

### DEGs related to iron uptake

Among the DGEs classified in COG P, a majority (38/66) of the genes were related to iron uptake, including 35 upregulated genes and 3 downregulated genes (Table 1). The 35 upregulated genes contained 9 genes encoding the TonB-dependent siderophore receptor, 6 genes encoding the TonB-dependent heme receptor, 5 genes encoding the TonB-ExbBD complex, 6 genes encoding proteins involved in biosynthesis of pyoverdine (the high-affinity siderophore of *P*. *fluorescens*) and some other genes related to heme uptake—such as heme receptor genes and the heme oxygenase gene *hemO* (HZ99_10895). HemO catalyzes the rate-limiting step in degrading heme to bilirubin and is essential for recycling iron from heme [44]. In addition, three genes that code for bacterioferritin were significantly downregulated. Bacterioferritins convert excess cellular iron (II) to iron (III), which is then stored as ferric-oxy-hydroxide-phosphate complexes [45]. According to the RNA-seq results, most of the genes in iron transport and metabolism pathways were significantly changed after EGCG exposure. The genes related to iron uptake can be induced by a series of environmental stresses, such as iron limitation [46], H_2_O_2_ stress [47], cold shock [48] or arsenic exposure [49]. Polyphenols are easily deprotonated at or below physiological pH (e.g., 5.0–8.0) in the presence of iron (III) and form complexes with stability constants comparable to iron-siderophore complexes [20, 50]. As *P*. *fluorescens* produces high-affinity siderophore pyoverdines in order to acquire iron, EGCG may play a role in inhibiting the pyoverdine-dependent transport of iron by competitive binding and may result in iron starvation. Moreover, some reports have indicated that green tea polyphenols or EGCG result in oxidative stresses in bacteria that depend on H_2_O_2_ release [19, 51]. For human tumor cell lines, the induction of apoptosis by EGCG was either partially or completely mediated by H_2_O_2_ [52]. In *Escherichia coli* H_2_O_2_ may antagonize the function of Fur protein, a major transcriptional repressor that controls ferric uptake, by oxidizing the Fur-Fe^2+^ complex and inactivating its repressor function [53], which then results in up regulation of these iron acquisition-related genes.

**Table 1:** DEGs related to iron uptake.

Gene	Log_2_FC	FDR	Gene product description
**Siderophore receptor**			
HZ99_12680	11.97	2.80E-13	TonB dependent siderophore (ferric enterobactin) receptor FepA
HZ99_15300	8.81	1.33E-05	TonB-dependent siderophore (ferrichrome) receptor
HZ99_00225	8.15	2.29E-04	TonB-dependent siderophore (ferric coprogen) receptor FhuE
HZ99_26610	7.87	7.03E-04	TonB-dependent siderophore (ferric coprogen) receptor FhuE
HZ99_18870	6.70	2.58E-02	TonB-dependent catecholate siderophore receptor CirA
HZ99_22610	6.14	2.94E-09	TonB-dependent ferric enterochelin receptor FepA
HZ99_18545	4.40	4.06E-05	TonB-dependent ferrichrome receptor
HZ99_00065	3.71	1.74E-04	ferric-pseudobactin BN7/BN8 receptor
HZ99_00780	2.64	2.08E-02	TonB-dependent ferric coprogen receptor FhuE
**Heme receptor**			
HZ99_10890	8.64	2.92E-16	TonB-dependent heme/hemoglobin receptor
HZ99_24755	4.25	1.76E-05	TonB-dependent heme/hemoglobin receptor
HZ99_17300	2.96	7.48E-03	TonB-dependent heme/hemoglobin receptor
HZ99_13170	2.91	1.22E-03	TonB-dependent heme/hemoglobin receptor
HZ99_11600	2.72	2.40E-02	TonB-dependent heme/hemoglobin receptor
HZ99_02135	2.67	2.22E-02	TonB-dependent heme/hemoglobin receptor
**TonB-ExbBD complex**			
HZ99_01780	5.05	2.27E-07	Energy transducer TonB
HZ99_17025	4.74	8.96E-08	biopolymer transporter ExbD
HZ99_17030	4.65	1.47E-07	Energy transducer TonB
HZ99_17020	4.32	8.42E-07	TonB-system energizer ExbB
HZ99_18895	2.54	1.82E-02	Biopolymer transporter ExbB
**Pyoverdine biosynthesis**			
HZ99_27755	9.55	2.66E-07	diaminobutyrate—2-oxoglutarate aminotransferase PvdH
HZ99_00255	9.05	3.50E-06	Chromophore maturation protein PvdO
HZ99_02660	8.69	2.23E-05	Acyl-homoserine lactone acylase PvdQ
HZ99_00250	7.39	4.21E-13	Pyoverdine ABC transporter, permease/ATP-binding protein PvdE
HZ99_27705	6.77	4.14E-13	Non-ribosomal peptide synthetase PvdL
HZ99_00290	6.66	4.01E-11	L-ornithine 5-monooxygenase PvdA
HZ99_00220	6.10	2.40E-11	Pyoverdine sidechain peptide synthetase IV
**Other iron uptake related genes**			
HZ99_10895	11.21	2.08E-11	Heme oxygenase HemO
HZ99_27680	10.23	6.28E-09	Acetyltransferase, siderophore biosynthesis protein
HZ99_22370	8.15	2.29E-04	Substrate-binding protein function in the ABC transport of ferric siderophores
HZ99_02655	7.28	5.74E-03	Fe^2+^ Zn^2+^ uptake regulation protein
HZ99_10340	4.14	2.23E-06	Imelysin-like iron-regulated protein A-like
HZ99_16430	2.14	2.08E-02	Iron ABC transporter substrate-binding protein
HZ99_23135	2.12	2.56E-02	TonB-dependent ferric citrate transporter FecA
**Iron storage**			
HZ99_11350	3.66	4.06E-05	(2Fe-2S)-binding protein
HZ99_11355	-5.86	7.06E-11	Bacterioferritin
HZ99_20890	-3.41	1.96E-04	Bacterioferritin
HZ99_14300	-2.96	8.43E-04	Bacterioferritin

### DEGs related to antioxidation and DNA repair

Transcriptional changes were also observed for genes that encode oxidative stress response proteins, such as superoxide dismutases, which counter reactive oxygen species by converting O_2_^-^ to H_2_O_2_ (Table 2). The *sodA* gene (HZ99_12430) that encodes manganese-based superoxide dismutase was markedly upregulated after EGCG exposure. *sodA* is part of the HZ99_12445-*fumC*- HZ99_12435-*sodA* operon, which is inducible by treatment with H_2_O_2_ or iron starvation, as well as Fur repression [46–47]. Based on the RNA-seq data, the other two genes in the operon were also significantly upregulated (S4 Table). Conversely, the *sodB* gene (HZ99_10325), which codes for a superoxide dismutase that utilizes iron as cofactor, was significantly downregulated after EGCG treatment (Table 2), presumably due to reduced iron availability in the cells after EGCG treatment. Other relevant for oxidative stress adaptation genes were also upregulated, including genes coding for alkylhydroperoxidase, peroxiredoxin, and AhpF (alkylhydroperoxide reductase subunit F) (Table 2). The *ahpF* gene has been induced by treatment with H_2_O_2_ [47, 54]. These genes are important to bacterial defense against toxic peroxides and were significantly upregulated in *P*. *fluorescens* after EGCG treatment, suggesting a cell response mediated by EGCG-induced oxidative stress.

**Table 2:** DEGs related to antioxidation and DNA repair.

Gene	Log_2_FC	FDR	Gene product description
**Antioxidation**			
HZ99_12430	7.07	1.49E-12	superoxide dismutase SodA
HZ99_06790	6.44	4.74E-02	alkylhydroperoxidase
HZ99_27730	2.43	1.51E-02	peroxiredoxin
HZ99_02330	2.21	2.80E-02	alkylhydroperoxide reductase subunit F, AhpF
HZ99_10325	-3.91	5.61E-06	superoxide dismutase SodB
**DNA repair**			
HZ99_25720	7.94	5.77E-04	ATP-dependent DNA helicase RecQ
HZ99_25700	7.28	5.74E-03	recombinase RmuC
HZ99_11700	7.20	7.42E-03	DNA repair protein RecO
HZ99_18100	6.58	3.49E-02	DNA repair photolyase SplB
HZ99_13290	4.21	4.29E-05	endonuclease
HZ99_01050	3.29	1.36E-02	exonuclease subunit SbcC
HZ99_24920	2.44	1.44E-02	uracil-DNA glycosylase UDG
HZ99_16060	2.17	2.89E-02	DNA-formamidopyrimidine glycosylase MutM
HZ99_22865	-7.61	1.87E-03	deoxyribodipyrimidine photolyase Phr

In addition to impact of EGCG on the gene transcription of antioxidant enzymes, there was an increase in mRNA levels of 8 genes relevant for DNA repair in EGCG-treated cells (Table 2). Among these upregulated genes, the *recQ* (HZ99_25720) and *rmuC* (HZ99_25700) were observed to belong to the SOS regulon in *E*. *coli* [55–56]. The SOS response is induced by DNA-damaging factors, such as UV, oxidants and antibiotics [57]. The *recO* gene encodes a gap repair protein and is essential for processing DNA damage prior to SOS induction in *E*. *coli* [58]. Consistent with the present results, previous research has demonstrated that green tea polyphenols also induced two DNA repair-related genes, *lexA* and *recN*, in *P*. *aeruginosa* [19]. Increased expression of genes related to DNA repair implies that EGCG caused DNA damage and induced the DNA damage response in *P*. *fluorescens*.

### DEGs related to cell envelope and cell-surface component synthesis

Several genes involved in cell envelope biogenesis and polysaccharide synthesis—in addition to other surface components—were differentially expressed in *P*. *fluorescens* after EGCG exposure (Table 3). Among the 24 DEGs that encode proteins in group COG M, 7 upregulated genes were related to lipopolysaccharide biosynthesis and 4 upregulated genes were related to peptidoglycan biosynthesis (Table 3). For example, the *wzzB* gene (HZ99_27115) encodes a lipopolysaccharide O-antigen chain length determinant protein which confers resistance to colicin E2 and the serum complement [59–60]. The *rfbC* gene (HZ99_27125) codes for an enzyme that reduces the lethal effects of antimicrobial agents and environmental stressors in *E*. *coli* [61]. The peptidoglycan biosynthesis protein MviN, encoded by HZ99_00070, is required for cell viability and maintenance of cellular shape and integrity [62–63]. The expression of *murF* (HZ99_12130), which encodes for an enzyme that catalyzes the last cytoplasmic step in peptidoglycan biosynthesis, was upregulated in response to subinhibitory doses of EGCG in *Staphylococcus aureus* [28]. In addition to DGEs related to lipopolysaccharide and peptidoglycan biosynthesis, a number of genes related to envelope lipoproteins (MlaA, RlpA), outer membrane porins (OprB, OprD), and exopolysaccharides (PgaB, WcaA, AlgL) were also differentially expressed after EGCG exposure. The expression of genes with functions related to cell wall synthesis and maintenance was affected by external stresses that damaged cell envelope, such as antibiotics that specifically target the cell envelope [64]. Some reports have demonstrated that catechins inhibit bacterial growth by affecting the structure and function of the cell wall [14, 65]. In this study, EGCG acted as a cell wall stress signal that induced the transcription of genes that encode cell wall repair enzymes.

**Table 3:** DEGs related to cell envelope and cell-surface component synthesis.

Gene	Log_2_FC	FDR	Gene product description
**Lipopolysaccharide**			
HZ99_27115	7.81	8.42E-04	LPS O-antigen chain length determinant protein WzzB
HZ99_25755	6.92	1.53E-02	UDP-2,3-diacylglucosamine hydrolase LpxH
HZ99_27125	6.70	2.58E-02	dTDP-4-dehydrorhamnose 3,5-epimerase RfbC
HZ99_19685	3.43	1.18E-03	4-amino-4-deoxy-L-arabinose transferase ArnT
HZ99_00100	3.57	7.76E-04	glycosyl transferase family 1 RfaB
HZ99_00085	2.50	3.07E-02	glycosyl transferase family 1 RfaB
HZ99_12380	2.24	3.63E-02	3-deoxy-D-manno-octulosonate 8-phosphate phosphatase KdsC
HZ99_22965	-3.22	3.05E-03	glycoside hydrolase RfaB
**Peptidoglycan**			
HZ99_00070	7.32	4.53E-03	peptidoglycan biosynthesis protein MviN
HZ99_13545	3.56	1.05E-02	LD-carboxypeptidase
HZ99_17745	2.42	7.97E-03	glucosamine fructose-6-phosphate aminotransferase GlmS
HZ99_12130	1.98	4.14E-02	UDP-N-acetylmuramoyl-tripeptide—D-alanyl-D-alanine ligase MurF
HZ99_06200	-2.36	2.52E-02	D-alanyl-D-alanine endopeptidase PBP7
**Cell wall related proteins and exopolysaccharides**			
HZ99_06855	8.84	1.03E-05	porin OprD
HZ99_18605	7.28	5.74E-03	poly-beta-1,6-N-acetyl-D-glucosamine N-deacetylase PgaB
HZ99_20290	7.11	9.43E-03	glycosyl transferase WcaA
HZ99_16630	6.70	2.58E-02	small-conductance mechanosensitive channel MscS
HZ99_08015	5.00	1.44E-03	lipoprotein component MlaA
HZ99_12515	-2.53	1.87E-02	rare lipoprotein A RlpA
HZ99_17495	-3.02	1.84E-03	glycosyl transferase WcaA
HZ99_04930	-6.50	4.74E-02	mechanosensitive ion channel protein MscS
HZ99_11950	-8.10	2.78E-04	poly(beta-D-mannuronate) lyase AlgL
HZ99_11605	-8.46	5.54E-05	porin OprB
HZ99_19350	-8.94	6.09E-06	choline transporter

### DGEs related to efflux

In the functional group COG V, there were 14 genes (82.35%) that encode efflux system proteins, including 13 upregulated genes and 1 downregulated gene (Table 4). Efflux is a mechanism that is responsible for bacterial antibiotic resistance. Active drug efflux is ascribed to low intrinsic susceptibility, cross-resistance to chemically unrelated classes of molecules, and selection/acquisition of additional resistance mechanisms [66]. Among the 12 upregulated efflux transporters, there were 6 Resistance-Nodulation-Cell Division (RND)-type multidrug efflux pumps. The expression of RND pumps is regulated in response to external stress factors—such as reactive oxygen species—or other agents that impose stress to the bacterial cell, like membrane damaging agents or ribosome blocking substances [67]. Thus, efflux pumps may be a component of a versatile protection mechanism against cellular stresses from EGCG exposure.

**Table 4:** DGEs related to efflux system.

Gene	Log_2_FC	FDR	Gene product description
**Efflux system**			
HZ99_18530	8.02	3.99E-04	RND efflux transporter NodT
HZ99_18585	5.34	3.62E-08	RND efflux transporter NodT
HZ99_03985	5.17	7.91E-04	MexE family multidrug efflux RND transporter periplasmic adaptor subunit
HZ99_03845	4.43	4.88E-06	RND efflux transporter NodT
HZ99_12050	3.88	3.06E-03	RND efflux transporter MFP subunit
HZ99_00280	3.48	1.66E-04	MacB family efflux pump subunit
HZ99_10930	3.45	1.09E-03	MexH family multidrug efflux RND transporter periplasmic adaptor subunit
HZ99_03855	2.86	1.79E-03	multidrug efflux system subunit MdtB
HZ99_08195	2.69	1.98E-02	MATE multidrug family efflux transporter
HZ99_05915	2.44	8.34E-03	MFS efflux transporter EmrB
HZ99_03850	2.35	1.22E-02	multidrug efflux system subunit MdtC
HZ99_24615	2.24	1.26E-02	multidrug efflux system transporter AcrA
HZ99_24610	1.92	3.77E-02	multidrug efflux RND transporter permease subunit
HZ99_18980	-2.71	2.90E-03	Multidrug efflux pump subunit AcrB

### DEGs related to metabolism and energy production

EGCG exposure in *P*. *fluorescens* caused the expression of a large group of genes that are related to bacterial metabolism (S4 and S5 Tables). This was especially notable in genes classified to COGs E (26 genes), G (19 genes), I (18 genes) and C (32 genes). Many group E and G genes encode enzymes and components of various transport systems. In group E, a majority of the genes (20/29) associated with amino acid transport and metabolism were upregulated, and, among them, there were 4 genes (HZ99_19340, HZ99_24080, HZ99_19490 and HZ99_18995) involved in the transport and metabolism of sulfur amino acids, cysteine and methionine. In the case of group G, the expression of some genes for sugar transport systems was significantly upregulated after EGCG exposure, such as HZ99_03895 that codes for glycerol-3-phosphate transporter UgpC and HZ99_20215 that codes for predicted arabinose efflux permease AraJ. Interestingly, in group I two *fabG* genes (HZ99_21295 and HZ99_02630) that encode type II fatty-acid synthases were remarkably upregulated. The *fabG* gene was observed to be induced by heat, oxidative damage, hypoxia and/or nutrient starvation stressors [68–69]. Among COG C, a majority of the genes (18/32) were downregulated, including nine respiratory chain genes related to cytochrome *c* oxidase and 3 genes related to nitrate reductase. Similar expression changes were also observed when *P*. *fluorescens* Pf-5 was under limiting iron conditions [46]. The changes observed in the expression of groups G, E, I and C may suggest metabolism response and adjustment to EGCG stress.

### DGEs related to transcription and signal transduction mechanisms

Bacterial adaptation to environmental stimuli is mediated primarily through transcription and signal transduction. Based on the RNA-seq results, the functional group COG K was also highly represented in *P*. *fluorescens* after EGCG exposure (Fig 4), while a significant majority (30/41) of the genes from COG K were upregulated. These DGEs encoded transcriptional factors from various families, including 10 alternative sigma factors (Table 5), 4 anti-sigma factors, 6 LysR family transcriptional regulators, 2 XRE family transcriptional regulators, 4 OmpR family DNA-binding response regulators, 3 MarR family transcriptional regulators, 3 AraC family transcriptional regulators, 1 TetR family transcriptional regulator, and 1 Fis family transcriptional regulator (S4 and S5 Tables). Alternative sigma factors control a wide variety of adaptive responses to environmental stresses [70]. Putative functions were assigned for HZ99_27685, HZ99_23165, HZ99_17290 and HZ99_24995 based on sequence conservation. HZ99_27685 encodes the extra-cytoplasmic function (ECF) sigma factor PvdS, a transcriptional regulator of pyoverdine biosynthesis genes [71]. The upregulation of *pvdS* is in agreement with increased expression of some pyoverdine biosynthesis genes (Table 1). PvdS has considerable similarity to the *E*. *coli* sigma factor FecI, a positive regulator of genes involved in ferric citrate transport, and FecI is controlled by the anti-sigma factor FecR, an inner-membrane sensor that transducers signals to FecI [72]. Accordingly, the RNA-seq data indicated that 5 genes coding for FecR were also highly upregulated. It was reported that *pvdS* and *fecR* were regulated by the Fur repressor [47, 73], suggesting adaptation responses to the iron limitation or oxidative stresses caused by EGCG. In addition, two *rpoE* genes (HZ99_23165 and HZ99_17290) were significantly upregulated by EGCG treatment, which was consistent with previous reports that antibiotics targeting the cell envelope induce a generalized response that involves activation of transcription of RpoE, as well as elements of heat, osmotic and oxidative stress regulons [64]. The RpoE regulon contains cell wall biogenesis genes, lipid detoxification gene *ahpF* and DNA repair related genes in *E*. *coli* K-12 [74] and RpoE has been characterized to be an important regulatory factors in response to environmental stress, especially envelope stress [70]. Thus, the two *rpoE* genes of *P*. *fluorescens* might be induced by envelope stress that is caused by EGCG. As shown in Tables 2 and 3, the expression of genes involved in cell wall biogenesis, oxidative stress and DNA damage was altered after EGCG exposure, which may be dependent on RpoE. Among the ten sigma factor genes, only the gene HZ99_24995, which encodes ECF sigma factor AlgU, was significantly downregulated after EGCG exposure, which mainly controls expression of genes for alginate biosynthesis. The decreased expression of *algU* was consistent with the observation that *algL* expression (HZ99_11950), an alginate biosynthesis related gene, was obviously downregulated after EGCG treatment (Table 3). In addition to the alternative sigma factors, many other genes that encode transcriptional regulators in different families also responded to EGCG exposure. However, a majority of these regulators were putative DNA-binding transcriptional regulators with undetermined functions.

**Table 5:** DGEs related to alternative sigma factors and two-component system histidine kinases.

Gene	Log_2_FC	FDR	Gene product description
**Alternative sigma factors**			
HZ99_02125	11.97	2.80E-13	RNA polymerase sigma factor
HZ99_18535	7.63	8.90E-12	RNA polymerase sigma factor
HZ99_27685	7.33	2.63E-13	RNA polymerase sigma factor, ECF subfamily, PvdS
HZ99_23165	5.35	7.59E-08	RNA polymerase subunit sigma-24, ECF subfamily, RpoE
HZ99_15290	4.92	3.17E-07	RNA polymerase sigma factor
HZ99_23145	4.75	1.96E-06	RNA polymerase sigma factor
HZ99_13180	3.71	5.54E-05	RNA polymerase sigma, ECF subfamily
HZ99_17290	2.87	8.65E-03	RNA polymerase sigma-24 factor, ECF subfamily, RpoE
HZ99_24765	2.64	4.90E-03	RNA polymerase sigma factor
HZ99_24995	-4.31	9.67E-05	RNA polymerase sigma-H factor AlgU, ECF subfamily
**Two-component system histidine kinase**			
HZ99_10445	8.29	5.53E-11	two-component sensor histidine kinase BaeS
HZ99_14705	7.87	7.03E-04	histidine kinase
HZ99_01210	7.36	4.53E-03	two-component sensor histidine kinase RstB
HZ99_27235	6.92	1.53E-02	K^+^-sensing histidine kinase KdpD
HZ99_22560	2.87	1.36E-02	two-component sensor histidine kinase
HZ99_23875	2.24	4.64E-02	histidine kinase EvgS
HZ99_25705	2.24	4.64E-02	histidine kinase
HZ99_23310	-4.40	2.14E-05	histidine kinase
HZ99_15325	-4.55	4.46E-02	two-component system sensor histidine kinase CreC
HZ99_22295	-5.60	5.54E-05	histidine kinase
HZ99_02845	-6.72	2.58E-02	two-component sensor histidine kinase
HZ99_00900	-7.30	4.53E-03	hybrid sensor histidine kinase/response regulator BaeS
HZ99_11515	-8.23	1.42E-04	two-component system response regulator RpfG

Importantly, in the COG T group, there were 35 genes differentially expressed after EGCG treatment, including two-component system genes, second messenger system genes and chemotaxis protein (S4 and S5 Tables). Among these DGEs, there were 13 genes that coded for two-component system histidine kinases (Table 5), which play a significant role in signaling transduction with transcriptional factors. For example, the highly upregulated gene HZ99_10445 encodes the two-component sensor histidine kinase BaeS, which is involved in envelope stress response in *E*. *coli*, while the main function of the Bae response is to upregulate efflux pump expression in response to specific envelope-damaging agents [75]. HZ99_10440, a COG R gene that codes for a LTXXQ domain protein, was adjacent to HZ99_10445 and was also highly upregulated after EGCG exposure (S4 Table). The two genes may be transcribed from the same operon, but the function of HZ99_10440 is still undetermined. HZ99_11515 codes for the two-component system response regulator RpfG, which was significantly downregulated. Mutation of *rpfG* greatly upregulates the genes related to extracellular polysaccharide production, such as *pgaB* in *Xanthomonas oryzae* pv. Oryzicola [76]. In this study, the *pgaB* gene (HZ99_18605) was observed to be upregulated (Table 3), which is in agreement with the downregulation of *rpfG*.

### Conclusions

In this study, for the first time transcriptomic analysis of the responses of *P*. *fluorescens* to EGCG was performed using RNA-seq technology. Expression of most DGEs involved in iron uptake, antioxidation, DNA repair, cell envelope and cell-surface component synthesis, and efflux system were significantly upregulated after EGCG exposure, while most genes associated with energy production were downregulated. These transcriptomic changes could be adaptive responses of *P*. *fluorescens* to iron limitation, oxidative stress, DNA and envelope damage that are caused by EGCG. In addition, many of the observed DGEs were ascribed to transcription and signal transduction mechanisms. Among them, the expression of specific genes encoding ECF sigma factor (PvdS, RpoE and AlgU) and two-component sensor histidine kinase (BaeS and RpfG) were strongly changed by EGCG treatment, and the related gene products may play important roles in regulating the stress responses of *P*. *fluorescens* to EGCG. The data presented in this study provides interesting insights into the molecular action related to the possible cross-resistance mediated by EGCG on the typical spoilage bacterium, *P*. *fluorescens*.

## Supporting information

S1 Fig Differentially-expressed genes of P. fluorescens exposed to EGCG. Red dots indicate differentially-expressed genes, while black dots represent genes that are not differentially expressed. The X-axis represents the average count of reads per million reads based on a log2 scale, while the Y-axis shows the fold-change values between the control and EGCG group based on a log2 scale. The blue horizontal line indicates the location at which the fold change is 2.
(TIF)

S1 Table Summary of reads in *P*. *fluorescens* transcriptome sequencing. (DOCX)

S2 Table Statistical results of clean reads mapping to the reference genome. (DOCX)

S3 Table List of primers used for RT-qPCR. (DOCX)

S4 Table All data related to the 291 upregulated genes. (XLSX)

S5 Table All data related to the 134 downregulated genes. (XLSX)
