# Supplementary figures and images for: Transcriptomic analysis of the response of Pseudomonas fluorescens to epigallocatechin gallate by RNA-seq
Source: PLoS One. 2017 May 17;12(5):e0177938. doi: 10.1371/journal.pone.0177938 (PMC5435343; doi:10.1371/journal.pone.0177938)

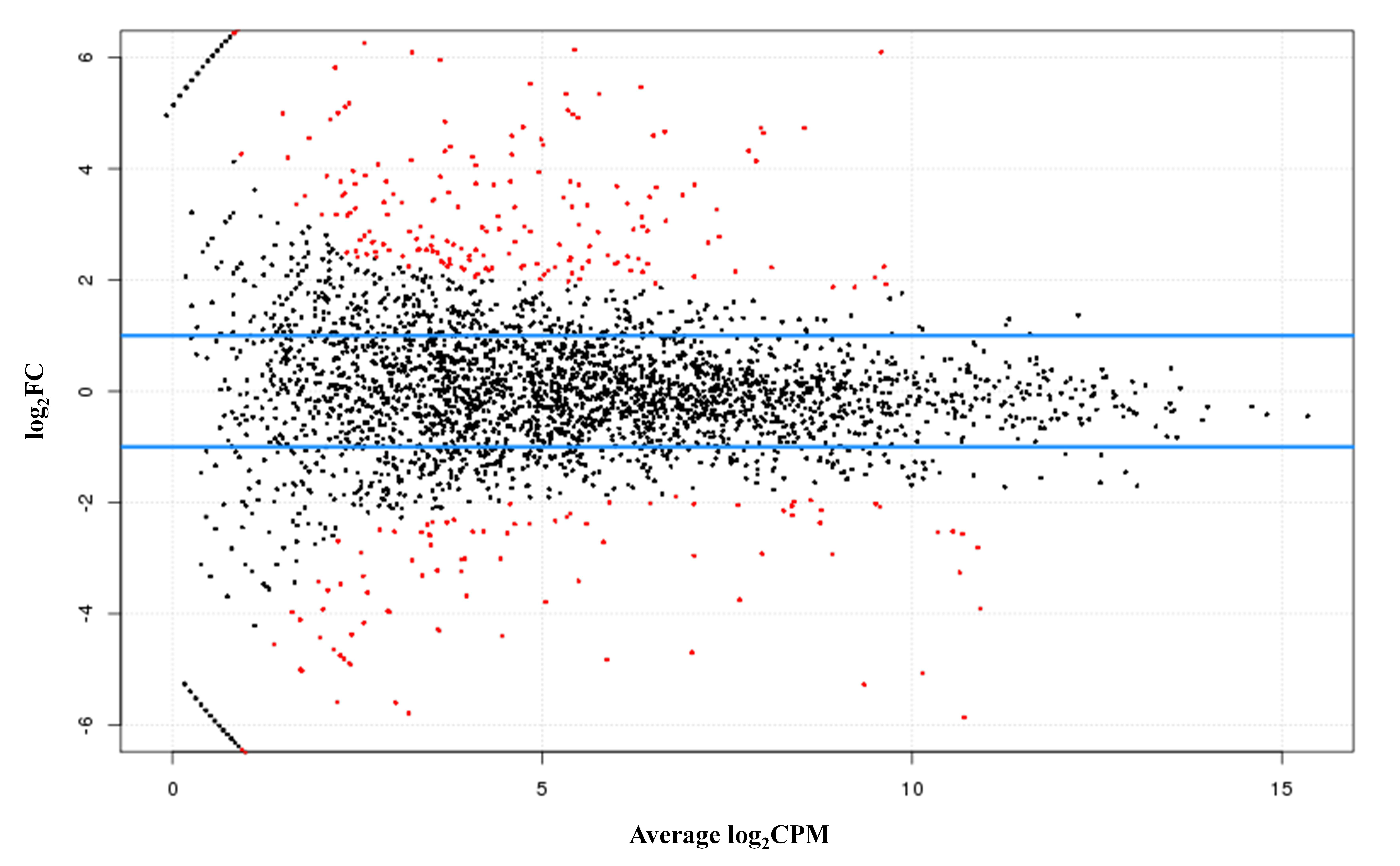

Supplement: S1 Fig — Red dots indicate differentially-expressed genes, while black dots represent genes that are not differentially expressed. The X-axis represents the average count of reads per million reads based on a log2 scale, while the Y-axis shows the fold-change values between the control and EGCG group based on a log2 scale. The blue horizontal line indicates the location at which the fold change is 2. (TIF) [file pone.0177938.s001.tif]
