# Supplementary material for: Transcriptomic analysis of the response of Pseudomonas fluorescens to epigallocatechin gallate by RNA-seq
Source: PLoS One. 2017 May 17;12(5):e0177938. doi: 10.1371/journal.pone.0177938 (PMC5435343; doi:10.1371/journal.pone.0177938)
Supplement: S1 Table — (DOCX) [file pone.0177938.s002.docx]

**S1 Table. Summary of reads in *P*. *fluorescens* transcriptome sequencing.**

| Sample | Clean reads | Read length | Clean bases | Q20 (%) | Q30 (%) | GC (%) |
| --- | --- | --- | --- | --- | --- | --- |
| Control-1 | 13182707 | 125 | 1.65 G | 92.68 | 85.68 | 56 |
| Control-2 | 13182707 | 125 | 1.65 G | 93.66 | 87.80 | 55 |
| Control-total | 26365414 | 125 | 3.30 G |  |  |  |
| EGCG-1 | 11643546 | 125 | 1.46 G | 93.31 | 86.81 | 56 |
| EGCG-2 | 11643546 | 125 | 1.46 G | 94.47 | 89.25 | 55 |
| EGCG-total | 23287092 | 125 | 2.92 G |  |  |  |

1: Reads sequenced from the left.

2: Reads sequenced form the right.

Q20, Q30: The percentage of bases with a phred value >20 or 30.

GC: GC content.
