# Supplementary material for: Transcriptomic analysis of the response of Pseudomonas fluorescens to epigallocatechin gallate by RNA-seq
Source: PLoS One. 2017 May 17;12(5):e0177938. doi: 10.1371/journal.pone.0177938 (PMC5435343; doi:10.1371/journal.pone.0177938)
Supplement: S2 Table — (DOCX) [file pone.0177938.s003.docx]

**S2 Table. Statistical results of clean reads mapping with reference genome**

| Map to genome | Control | | EGCG | |
| --- | --- | --- | --- | --- |
|  | Numbers | Percentage | Numbers | Percentage |
| Total reads | 26365414 |  | 23287092 |  |
| Total mapped | 15022386 | 57.00% | 13506803 | 58.00% |
| Uniquely mapped | 14970331 | 56.80% | 13450317 | 57.80% |
| Multiple mapped | 52055 | 0.20% | 56486 | 0.20% |
| Total gene count (reference genome) | 5178 |  | 5178 |  |
| Gene count (Reads ≥ 1 ) | 3180 | 61.41% | 3240 | 62.57% |
