# Supplementary material for: Transcriptomic analysis of the response of Pseudomonas fluorescens to epigallocatechin gallate by RNA-seq
Source: PLoS One. 2017 May 17;12(5):e0177938. doi: 10.1371/journal.pone.0177938 (PMC5435343; doi:10.1371/journal.pone.0177938)
Supplement: S3 Table — (DOCX) [file pone.0177938.s004.docx]

**S3 Table. List of primers used for RT-qPCR**

| Gene | Gene product description | Primer Sequences (5'to3') | Size (bp) |
| --- | --- | --- | --- |
| HZ99_12440 | class II fumarate hydratase FumC | F: CAGTGTCCTGGGAGCCAATCA | 103 |
|  |  | R: CCAGAATTCGCTGCACGTTT |  |
| HZ99_02125 | RNA polymerase sigma factor | F: GCCATTGAACGGGCCTACCT | 94 |
|  |  | R: GCAGGGTTTCGATCATCAGGTAA |  |
| HZ99_12680 | TonB dependent siderophore (ferric enterobactin) receptor FepA | F:GAGCAAACGCACCAACTTCAA | 94 |
|  |  | R: GTCGTCGGCATCGGTCTTGT |  |
| HZ99_12445 | FagA protein | F: CGCCCTTCGTTTAGGTAATGTTC | 80 |
|  |  | R: CGCTGGATGAGTCGCCAGATT |  |
| HZ99_10895 | heme oxygenase HemO | F: CAAGCTCGACGCACTGGTCA | 70 |
|  |  | R: GACGACAAAACGGGCGAAGTT |  |
| HZ99_00285 | hemolysin secretion protein D | F: GGGCAGTCTGCACGTCTTCAT | 138 |
|  |  | R: CTCGTTACTCCATCGCCAACCT |  |
| HZ99_00785 | iron dicitrate transport regulator FecR | F: GCTCCTGGGACACGAGGAAG | 112 |
|  |  | R: CACGGCGAACTGGTGATTCA |  |
| HZ99_10890 | TonB-dependent heme/hemoglobin receptor | F: CCAACCTGAACTACCGCAAACT | 89 |
|  |  | R: GGTCATTTGTTGCAGGGTGATG |  |
| HZ99_10445 | two-component sensor histidine kinase BaeS | F: CAATACCACGTCGATGTGCAAG | 112 |
|  |  | R: CCTCGTGGCTCTCATGCTGT |  |
| HZ99_00250 | pyoverdine ABC transporter, permease/ATP-binding protein PvdE | F: CCAATCACCACAAAAAACAGCAT | 90 |
|  |  | R: CGACCATATCTGCGATACCCATA |  |
| HZ99_10440 | LTXXQ domain protein | F: GCAAGACCCTTATCGCTCTGATG | 175 |
|  |  | R: GTCAGGTCGAGCTGGCTGAAG |  |
| HZ99_12430 | superoxide dismutase SodA | F: CATCAATGGGCTGTCCTGGTT | 96 |
|  |  | R: CTGACTCGCTTTGGCAGTGGT |  |
| HZ99_27705 | non-ribosomal peptide synthetase PvdL | F: CTGTACCCGCAGGACATCGA | 92 |
|  |  | R: CCTTGGTCATTCACCGCAAAC |  |
| HZ99_00290 | L-ornithine 5-monooxygenase PvdA | F: CAGAGCGAGTTGCAGATTTCCT | 125 |
|  |  | R: GTGCCCAGGTTGATAAAGTCCA |  |
| HZ99_12425 | zinc transporter ZupT | F: CCTGCAACGCGATACCCAT | 102 |
|  |  | R: CCATCATTGCCCACAACATTC |  |
| HZ99_22610 | tonB-dependent ferric enterochelin receptor FepA | F: GTGAGCGGCAGTTGCTGGAT | 77 |
|  |  | R: GACACGCCAATCTCCTTGTTGA |  |
| HZ99_16345 | NAD(P)H-dependent FMN reductase | F: CTGTTCCAGGGCTTCGCTCAA | 91 |
|  |  | R: CGAGGACAGCCAGATCGCCTA |  |
| HZ99_19350 | choline transporter | F: GCTACTTCACGCCGTCCTTTG | 144 |
|  |  | R: CGTTGATGATCTGCTCCTTGGT |  |
| HZ99_10325 | superoxide dismutase SodB | F: GCCGACGGAAGTCTTGGTGA | 78 |
|  |  | R: GCATTGGCTGAAGCCATCAA |  |
| HZ99_24995 | RNA polymerase sigma-H factor AlgU | F: CCAAGACCCAGGTGGTTGTAAA | 80 |
|  |  | R: CGTCACAGGCTCAAGGAACAG |  |
| HZ99_04470 | nitrate reductase subunit beta NarH | F: GATCCGTGGGTAGCAGAAGATG | 76 |
|  |  | R: GGCTGCCCGTACAAGAAAATC |  |
| HZ99_11355 | bacterioferritin | F: GATCAAGCGCATCCTGTTCCT | 101 |
|  |  | R:CCAGGTCGCTGTTGAGCATTT |  |
| 16s | internal control | F: CTGTGTAGCGGTGAAATGCGTAGA | 90 |
|  |  | R: GCTTTCGCACCTCAGTGTCAGTA |  |
